# Image-guided intensity-modulated radiotherapy of prostate cancer: Analysis of interfractional errors and acute toxicity

**DOI:** 10.1007/s00066-015-0919-y

**Published:** 2015-11-06

**Authors:** Volker Rudat, A. Nour, M. Hammoud, A. Alaradi, A. Mohammed

**Affiliations:** Department of Radiation Oncology, Saad Specialist Hospital, 31952 Al Khobar, Saudi Arabia

**Keywords:** Prostate neoplasms, Image-guided radiotherapy, Intensity-modulated radiotherapy, Planning target volume, Fiducial markers, Prostataneoplasien, Bildgesteuerte Strahlentherapie, Intensitätsmodulierte Strahlentherapie, Planungszielvolumen, Röntgendichte Marker

## Abstract

**Purpose:**

The aim of the study was to estimate interfractional deviations in patient and prostate position, the impact of the frequency of online verification on the treatment margins, and to assess acute radiation reactions of high-dose external beam image-guided intensity-modulated radiotherapy (IG-IMRT) of localized prostate cancer.

**Patients and methods:**

IG-IMRT was performed by daily online verification of implanted fiducial prostate markers using a megavoltage electronic portal imaging device (EPID). A total of 1011 image-guided treatment fractions from 23 consecutive unselected prostate cancer patients were analyzed. The median total dose was 79.2 Gy (range 77.4–81.0 Gy). Acute radiation reactions were assessed weekly during radiotherapy using the Common Terminology Criteria for Adverse Events (CTCAE) v.4.03.

**Results:**

A relevant combined patient set-up and prostate motion population random error of 4–5 mm was observed. Compared to daily IGRT, image guidance every other day required an expansion of the CTV–PTV (clinical target volume–planning target volume) margin of 8.1, 6.6, and 4.1 mm in the longitudinal, vertical, and lateral directions, thereby, increasing the PTV by approximately 30–40 %. No grade 3 or 4 acute radiation reactions were observed with daily IG-IMRT.

**Conclusion:**

A high dose with surprisingly low acute toxicity can be applied with daily IG-IMRT using implanted fiducial prostate markers. Daily image guidance is clearly superior to image guidance every other fraction concerning adequate target coverage with minimal margins.

## Abstract (de)

**Ziel:**

Ziel der Studie war es, die interfraktionelle Variabilität der Patientenlagerung und Prostataposition, den Einfluss der Bildgebungsfrequenz und die akuten Strahlenreaktionen bei einer hochdosierten bildgesteuerten intensitätsmodulierten Strahlentherapie (IG-IMRT) des Prostatakarzinoms zu untersuchen.

**Methoden:**

IG-IMRT wurde durch tägliche Verifikation von implantierten röntgendichten Prostatamarkern mittels Megavolt-Bildgebung (“electronic portal imaging device”, EPID) und anschließender Patientenrepositionierung vor Strahlentherapie erreicht. Insgesamt 1011 bildgesteuerte Fraktionen von 23 nacheinander behandelten, unselektierten Patienten wurden analysiert. Die mediane Gesamtbehandlungsdosis betrug 79,2 Gy (Spanne 77,4–81,0 Gy). Akutreaktionen wurden wöchentlich mittels der Common Terminology Criteria for Adverse Events (CTCAE) v.4.03 erfasst.

**Ergebnisse:**

Der Zufallsfehler der Gesamtpopulation durch die Variabilität der Patientenlagerung und Prostataposition betrug 4–5 mm. Bildgesteuerte Strahlentherapie (IGRT) würde bei jeder zweiten Bestrahlungsfraktion im Vergleich zu täglicher IGRT eine Erweiterung des CTV(klinisches Zielvolumen)-PTV(Planungszielvolumen)-Sicherheitssaums von 8,1 mm, 6,6 mm und 4,1 mm in der longitudinalen, vertikalen und lateralen Ebene erfordern. Dies würde eine Vergrößerung des PTV von etwa 30–40 % bedeuten. Bei der IG-IMRT mit täglicher Bildgebung wurden in der vorliegenden Studie keine Grad-3- oder Grad-4-Akutreaktionen beobachtet.

**Schlussfolgerung:**

Bei einer IG-IMRT mit täglicher Verifikation von Prostatamarkern kann eine hohe Dosis mit überraschend geringer Akuttoxizität appliziert werden. Im Hinblick auf adäquate Tumorerfassung mit geringstmöglichen Sicherheitssäumen ist die tägliche IGRT einer IGRT bei jeder zweiten Bestrahlungsfraktion deutlich überlegen.

Several meta-analyses have shown that higher doses of radiotherapy improve the biochemical relapse-free survival of patients with organ-confined prostate cancer compared to those treated with conventional-dose radiotherapy [7, 22]. Higher radiation doses potentially increase the risk of acute and late radiation toxicity. In order to keep the risk of acute and late radiation toxicity as low as possible, the radiotherapeutic high-dose region should be as small as possible.

Early studies revealed a relevant prostate motion variability [20], evaluated the patient set-up variability without image guidance [15], and estimated the treatment margins for the combined error of both factors [17]. Image-guided radiotherapy (IGRT) and reverse planned intensity-modulated radiotherapy (IMRT) are current radiation techniques commonly used to minimize the high-dose region without compromising tumor coverage for the definitive radiotherapy of localized prostate cancer. IGRT reduces the high-dose volume by minimizing the required internal margin (IM) and set-up margin (SM), thereby, downsizing the planning target volume (PTV). IMRT reduces the high-dose volume by generating a dose distribution more conformal to the PTV compared to conventional three-dimensional conformal radiotherapy (3DCRT).

In this study, IGRT was achieved with daily online verification of implanted fiducial prostate markers using an electronic portal imaging device (EPID). The goal of the study was to assess prostate motion variability and patient set-up variability, and to estimate the safety margin to accommodate for the combined error of both factors. Furthermore, the impact of the frequency of the image guidance (every fraction versus every other fraction versus no image guidance) on the CTV–PTV margin was estimated. Acute radiation reactions were assessed weekly during radiotherapy to evaluate the tolerance to high radiation doses applied using daily IG-IMRT.

## Patients and methods

### Patient data and preparation for treatment planning

A total of 23 consecutive, unselected patients receiving definitive radiotherapy for localized prostate cancer between December 2013 and March 2015 were analyzed. The histopathological diagnosis was established by transrectal ultrasonography (TRUS)-guided biopsy. Usually 12 cores were taken per prostate. Prior to radiotherapy, all patients underwent implantation of three prostate gold markers (1.2 × 3.0 mm in size; Civco Medical Solutions, Coralville, IA, USA) into the prostate under TRUS guidance to enable image-guided radiotherapy. The three prostate gold markers were placed in the prostate base, mid-gland, and apex. After an interval of 3 days, the patients underwent a computed tomography (CT) scan and magnetic resonance imaging (MRI) in the supine position for radiotherapy planning. The CT simulation was performed without contrast medium using a 64-slice spiral CT scanner (Somatom Sensation 64, Siemens Healthcare, Erlangen, Germany) with a slice thickness of 3 mm. The MRI was performed using a 3 T MRI scanner (Magnetron Trio, Siemens Healthcare, Erlangen, Germany). The slice thickness was 2 mm. Five sequences (axial T1w, axial T2w, coronal T2 STIR, axial T1FS) were obtained before and three sequences (axial, sagittal, and coronal T1FS) after the application of contrast media. The MRI and CT images were electronically fused using the Auto-Register method of the syngo®-based Coherence Oncologist Workspace version 2.0.52 (Siemens Healthcare, Erlangen, Germany). The Auto-Register method uses a “(Normalized) Mutual Information” algorithm which is based on information theory (entropy). The target volumes were defined using the fused images. MRI images were used for the target volume definition in particular because the apex of the prostate can be better visualized using MRI compared to CT [13, 23]. For CT simulation and radiotherapy, patients were immobilized in supine position using a headrest, kneefix, and feetfix (Civco Medical Solutions, Coralville, IA, USA). The CT simulator and the linear accelerators were equipped with identical models of a carbon index tables and positioning devices. Patients were instructed to have a comfortably filled bladder and an empty rectum for their CT and MRI examinations and for each treatment appointment.

The study was approved by the local institutional ethical committee and conducted in accordance with the Helsinki Declaration in its current version.

### Image-guided radiotherapy

**Fig. 1:**
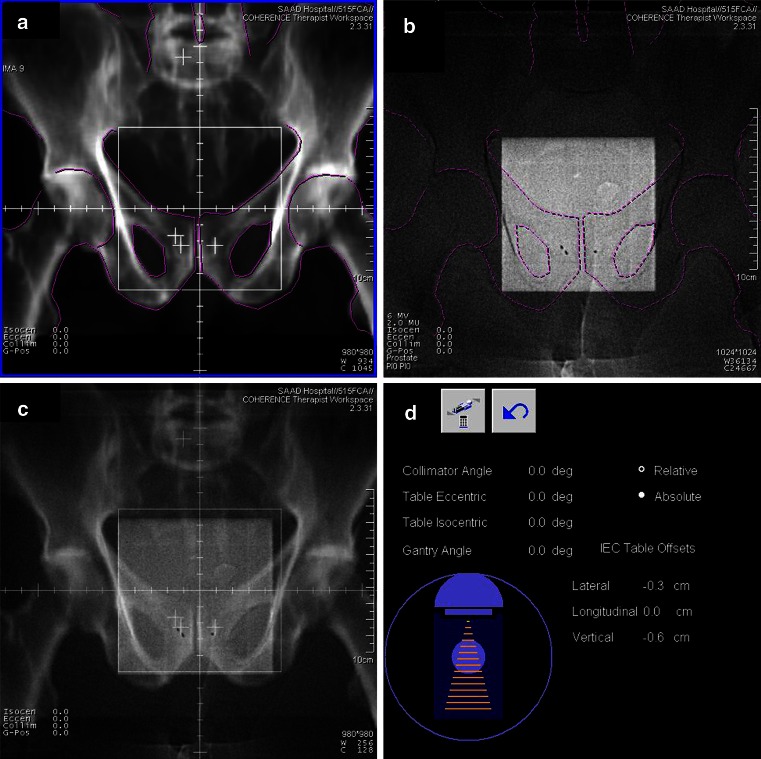
Online verification of bony landmarks and implanted fiducial prostate markers using megavoltage X-rays and electronic portal imaging device (*EPID*). **a** Digital reconstructed radiograph (*DRR*) generated by the treatment planning system. The position of the three fiducial prostate markers is marked with crosses. **b** Portal image (double-exposed) obtained immediately before the radiotherapy fraction using the EPID. The *three dots* represent the fiducial prostate markers. **c** Fused images of **a** and **b**. **d** Calculated deviation of the compared structures of images **a** and **b**

At the first radiotherapy fraction, megavoltage electronic portal images were taken using an electronic portal imaging device (EPID, [16]) from orthogonal directions (0° or 180° and 270° or 90°) and from the directions of the treatment beams. At all following radiotherapy fractions megavoltage electronic portal images were taken from two orthogonal directions. Processing and analysis software was used to significantly improve the image quality of the megavoltage electronic portal images [10]. Representative pelvic bony landmarks and the three fiducial prostate markers were marked on the portal images using electronic drawing tools. The images were zoomed and electronically superposed with the reference images, the corresponding digitally reconstructed radiographs (DRR) generated by the treatment planning system (TPS). A portal imaging software was used to assess the isocenter placement error in three dimensions based on the comparison of bony landmarks or fiducial prostate markers of the portal images with the corresponding reference image (Fig. 1).

The patient set-up error was represented by the deviation of the compared bony landmarks. The prostate motion error was represented by the deviation of the compared fiducial prostate markers after matching of the bony landmarks. The combined error of both factors (referred to as “combined error”) was represented by the deviation of the compared fiducial prostate markers. Online correction of deviation of the fiducial prostate markers was done by automatic adjustment of the treatment table in three dimensions prior to every radiotherapy application in all patients.

### Inverse-planned intensity-modulated radiotherapy

The target volumes were defined and the dose prescribed according to the International Commission on Radiation Units and Measurement (ICRU) Reports 50 and 62 recommendations. Accordingly, the PTV should be surrounded by the 95 % isodose line. The contouring of the PTV and organs at risk was done according to the RTOG Consensus Contouring Guidelines “Male Pelvis Normal Tissue” and other specific recommendations [13, 23]. In very low risk and low risk patients the CTV included the prostate gland. In one patient with very high risk, the CTV included the prostate, the complete seminal vesicals, and the locoregional pelvic lymph nodes. In all other patients the prostate gland and the proximal seminal vesicals were included in the CTV. The CTV to PTV margin for the prostate gland was 5 mm and for the seminal vesicals 8 mm. The IMRT plans were generated using the TPS XIO 4.4 (CMS, Inc., St. Louis, MO, U.S.A.). The normal tissue dose volume constraints provided by the TPS were used for the IMRT plans. Tissue inhomogeneities were considered in the treatment planning optimization process, and the dose calculation algorithm used was “superposition”. An optimization with 100 iterations was applied, followed by a semiautomatic segmentation (minimum 3 cm step size). Segments with less than or equal 2 MU were expelled from the plan. A step-and-shoot technique was used with usually eight equally spaced coplanar fields. The number of segments of a typical plan was around 100 and the corresponding treatment time about 15 min. Linear accelerators (Oncor Avant Garde, Siemens Medical, Erlangen, Germany) equipped with a multileaf collimator (160 leaves) and an EPID (Optivue, Siemens Medical, Erlangen Germany) were used for the treatment.

### Assessment of acute radiation reactions

Acute radiation reactions were prospectively assessed by two Radiation Oncologists using the Common Terminology Criteria for Adverse Events (CTCAE) v.4.03. The acute reactions were assessed once weekly during the course of radiotherapy and 6 weeks after radiotherapy.

## Statistical analysis

Individual and population based parameters of the patient set-up variability were calculated according to the report “On target: ensuring geometric accuracy in radiotherapy” by The Royal College of Radiologists [14]. Accordingly, the individual mean patient set-up error M_individual_ was defined as the mean set-up error for an individual patient. The overall population mean set-up error M_pop_ was defined as the overall mean for the analyzed patient group. The population systematic error Σ_set-up_ was defined as the standard deviation of the individual mean set-up error about the overall mean M_pop_. The individual random (daily) positioning error σ_individual_ was defined as the standard deviation of the set-up error around the corresponding mean individual value M_individual_. The population random error σ_set-up_ was defined as the mean of all individual random errors σ_individual_. The patient set-up parameters were calculated for each direction (longitudinal, vertical, and lateral).

Image-guided correction of the patient set-up and prostate motion error was performed prior to every radiotherapy fraction in all patients. In order to estimate the margins required for image guidance every other radiotherapy fraction or no image guidance during radiotherapy, the patient set-up and prostate motion error before correction of the corresponding radiotherapy fractions was used for the statistical analysis. Treatment margins were calculated using the van Herk formula [21]. Accordingly, the margin required to ensure 95 % minimum dose to the PTV for 90 % of the patients was given by

1 $$ {{M}_{ptv}}=2.50\sum +1.64\sigma -1.64{{\sigma }_{p}} $$

where Σ is the square root of the quadratic sum of the standard deviations of all contributing systematic errors, σ the square root of the quadratic sum of the standard deviations of all contributing random errors, and ${{\sigma }_{p}}$the standard deviation describing the width of the penumbra. In our analysis ${{\sum }_{set-up}}$was used as contributing systematic error, and ${{\sigma }_{set-up}}$ and ${{\sigma }_{p}}$as contributing random errors $(\sigma =\sqrt[2]{\sigma _{set-up}^{2}+\sigma _{p}^{2}})$. The representative standard deviation of the penumbra width ${{\sigma }_{p}}$of our linear accelerators was 4.2 mm.

## Results

**Fig. 2:**
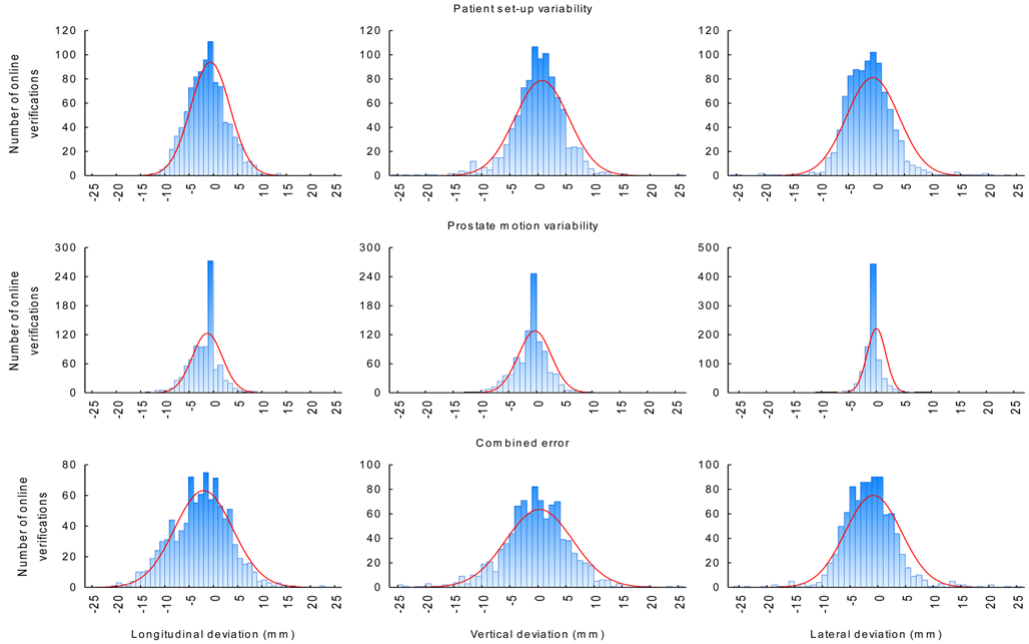
Patient set-up variability, prostate motion variability, and the combined error of both factors. The *red lines* represent the corresponding Gaussian distribution

**Fig. 3:**
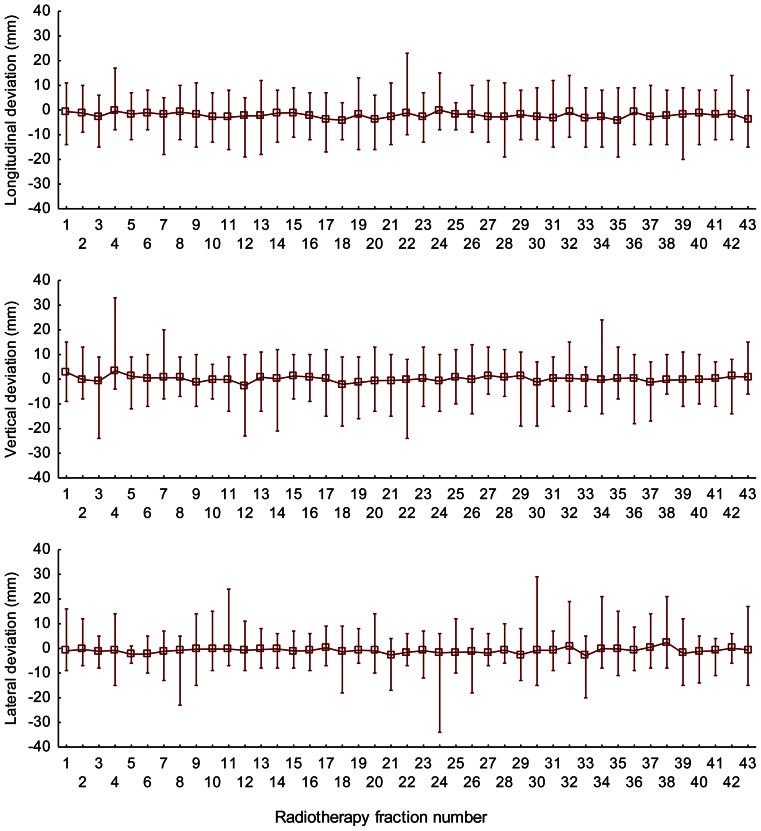
Combined error of the patient set-up variability and prostate motion variability during the course of radiotherapy. The *squares* represent the mean and the *vertical lines* the range of the deviation

**Fig. 4:**
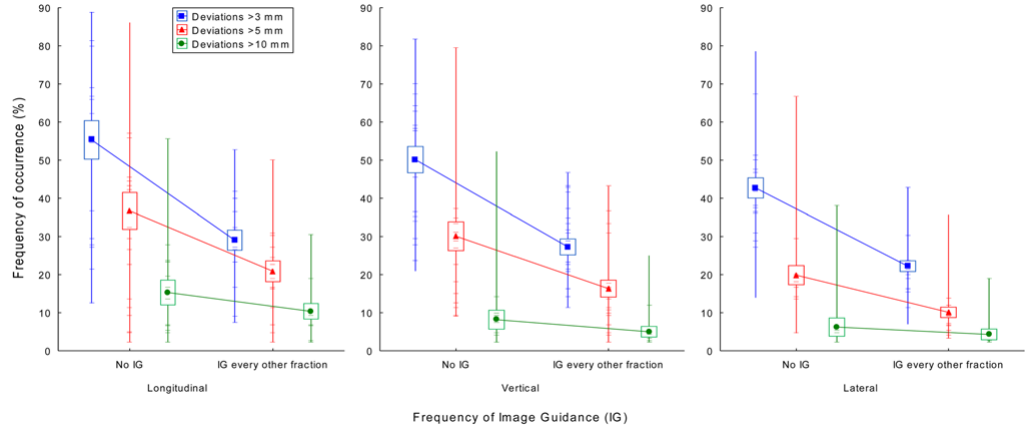
Frequency of the combined error of patient set-up variability and prostate motion variability larger than threshold. The *filled symbols* represent the population mean, the *box* the standard error, and the *horizontal lines* the mean of individual patients

A total of 1011 image-guided treatment fractions from 23 subsequent unselected prostate cancer patients were obtained for analysis. The number of radiotherapy fractions per patient ranged from 43 to 45. Patient and treatment characteristics are listed in Table 1. The patient set-up variability, prostate motion variability, and the combined error of both factors (referred to as combined error) were slightly different in the three dimensions. On average, the patient set-up variability was greater than the prostate motion variability. Most probably due to differences in the bladder and rectum filling [20, 21] and respiration [4] the prostate motion variability was greater in the longitudinal and vertical direction than in the lateral direction. All errors were compatible with a Gaussian distribution (Fig. 2). The mean combined error appeared to be constant during the course of the radiotherapy (Fig. 3). Image guidance every other fraction compared to no image guidance substantially reduced the combined error. However, considerable error remains after image guidance every other fraction. The mean combined error varied considerably between and within the patients, and depending on the direction deviations greater than 3, 5, and 10 mm were found on average in 22–29 %, 10–21 %, and 4–10 % of the radiotherapy fractions after image guidance every other fraction (Fig. 4). The patient set-up variability, prostate motion variability, and the required safety margin to accommodate for the combined error in dependence of the frequency of the image guidance are listed in Table 2. Intrafractional errors were not investigated in this study.

**Table 1:** Patient and treatment characteristics

Characteristics		*n*	%
Age (years)			
	41–50	1	4.3
	51–60	3	13.0
	61–70	7	30.4
	71–80	12	52.2
Body mass index			
	Normal weight	7	30.4
	Overweight	10	43.5
	Obese	6	26.1
T classification			
	T1	5	21.7
	T2	13	56.5
	T3	4	17.4
	T4	1	4.3
N classification			
	N0	23	100.0
M classification			
	M0	23	100.0
Risk group (NCCN guidelines)^a^			
	Very low	2	8.7
	Low	2	8.7
	Intermediate	6	26.1
	High	7	30.4
	Very high	6	26.1
Total dose (Gy)^b^			
	Median	79.2	
	Min–Max	77.4–81.0	
ADT^c^			
	None	4	17.4
	4–6 months	6	26.1
	2–3 years	13	56.5

*LN* Locoregional lymph nodes, *ADT* androgen deprivation therapy.

^a^Guidelines of the National Comprehensive Cancer Network.

^b^Dose per fraction was 1.8 Gy in all patients.

^c^ADT was given during radiotherapy.

**Table 2:** Patient set-up variability, prostate motion variability, and safety margin (mm) to accommodate for the combined error of both factors using the van Herk formula

		Patient set-up variability			Prostate motion variability			Combined error			
	Direction	M	Σ	σ	M	Σ	σ	M	Σ	σ	Safety margin^a^
No image guidance											
	Longitudinal	− 0.7	2.8	2.8	− 1.4	1.9	2.4	− 2.1	3.4	4.3	11.4
	Vertical	0.6	2.7	3.7	− 0.4	1.7	2.4	0.1	3.2	4.8	11.5
	Lateral	− 0.7	2.0	3.6	− 0.1	0.7	1.5	− 0.9	2.1	4.2	8.2
Image guidance every other fraction											
	Longitudinal	− 0.5	1.5	2.4	− 0.8	1.1	2.0	− 1.2	2.3	3.8	8.1
	Vertical	0.3	1.5	2.7	− 0.1	0.9	1.9	0.2	1.7	3.6	6.6
	Lateral	− 0.4	1.1	2.7	− 0.1	0.4	1.1	− 0.6	1.0	3.1	4.1

*M* Overall population mean set-up error, *Σ* Population systematic error, *σ* Population random error.

^a^Safety margin to accommodate for the combined error of patient set-up accuracy and prostate motion using the van Herk formula.

In two patients a migration of one of the three fiducial prostate markers was detected. In one patient a fiducial prostate marker migration was observed between the CT simulation and the first radiotherapy fraction. In another patient a migration of a fiducial prostate marker of 4 mm was observed at radiotherapy fraction number 37. A loss of a fiducial prostate marker was not observed.

Acute radiation reactions were assessed weekly throughout the course of radiotherapy and 6 weeks after radiotherapy using CTCAE v.4.03. Despite the high radiation dose of 77.4–81.0 Gy applied no grade 3 or 4 acute radiation reactions were observed. Grade 2 acute reactions were detected in 4.3–56.5 %, and grade 1 in 13.0–78.3 % of the patients (Table 3). It should be noted that the common acute reaction “urinary frequency” is categorized in CTCAE v. 4.03 as grade 0, grade 1 (“present”), and grade 2 (“limiting instrumental activities of daily living; medical management indicated”). Grade 3 or 4 has not been defined. In contrast, the Acute Radiation Morbidity Scoring Criteria of the Radiation Therapy Oncology Group (RTOG) categorizes genitourinary morbidity into grades 0–4. This should be considered if results are compared. However, only 2 patients (13 %) of our study population developed CTCAE v. 4.03 grade 2 urinary frequency (Table 3).

**Table 3:** Maximal acute radiation reactions [Common Terminology Criteria for Adverse Events (CTCAE) v.4.03]

Parameter	Grade	*n*	%
Dermatitis radiation			
	0	9	39.1
	1	13	56.5
	2	1	4.3
Diarrhea			
	0	20	97.0
	1	3	13.0
Fatigue			
	0	7	30.4
	1	13	56.5
	2	3	13.0
Gastrointestinal pain			
	1	10	43.5
	2	13	56.5
Proctitis			
	0	9	39.1
	1	13	56.5
	2	1	4.3
Urinary frequency			
	0	2	8.7
	1	18	78.3
	2	3	13.0
Weight loss			
	0	23	100.0

## Discussion

In our study, IGRT was achieved by daily online verification of implanted fiducial prostate markers. In order to save cost in terms of increased dose and in-room time [9], the question arises whether the frequency of image guidance can be reduced from daily to every other day without losing relevant benefit.

Our data show that the combined patient set-up and prostate motion error on average remains basically constant over the course of radiotherapy. A small number of image-guided treatment fractions at the beginning of the radiotherapy course should therefore be sufficient to significantly reduce the systematic error. However, our data also reveal a population random error of 4–5 mm in all directions, and that the random error varied grossly between and within the patients. For this reason, CTV–PTV margins derived from population-based observations would lead to unnecessarily large PTVs in many patients. The impact of the random error on the CTV–PTV margin can be significantly reduced by daily online verification of the prostate position with necessary corrections applied before delivery of treatment. Our data show that with this approach in combination with the use of IMRT high doses of 77.4–81.0 Gy can be delivered with surprisingly low acute radiation toxicity. According to the van Herk formula, reducing the frequency of image-guided fractions to every other fraction would require an expansion of the CTV–PTV margin of 4–8 mm. This additional margin would increase the PTV by approximately 30–40 % in a typical prostate cancer patient. It is likely that an increase of the PTV of this magnitude will significantly increase the risk of toxicity at the high radiation doses prescribed.

Similar results have been reported by Kupelian et al. [11]. In their study, prostate cancer patients were treated with helical tomotherapy and megavoltage computed tomography images were used for image guidance with intraprostatic metallic fiducials. In agreement with our study, imaged guidance every other day compared to daily image guidance would have increased the CTV–PTV margin by 4–7 mm using the van Herk formula. The authors concluded that high-dose external beam radiotherapy for localized prostate cancer delivered with tight treatment margins requires daily image guidance.

Clinical data concerning toxicity of IG-IMRT in daily practice are scarce. Takeda et al. [19] reported about 141 patients with localized prostate cancer treated with IG-IMRT to a total dose of 76 Gy (*n* = 13) and 80 Gy (*n* = 128). No grade 3 or 4 acute toxicities were observed. The incidence of grade 2 acute gastrointestinal (GI) and genitourinary (GU) toxicities were 1.4 and 8.5 %, respectively. The 5-year actuarial likelihood of grade 2–3 GI and GU late toxicities were 6 and 6.3 %, respectively. There was no grade 4 GI or GU late toxicity. Wortel et al. [25] assessed the acute radiation toxicity of patients treated to 78 Gy with either IG-IMRT (*n* = 260) or 3D-CRT (*n* = 215) using toxicity questionnaires distributed at baseline, prior to fraction 20 and 30, and at 90 days after treatment. IG-IMRT resulted in significantly lower overall RTOG grade 2 or higher GI toxicity (29 versus 49 %, respectively, *p* = 0.002) and overall GU grade 2 and higher toxicity (38 versus 48 %, respectively, *p* = 0.009). Michalski et al. [12] compared 491 patients treated with 3D-CRT and 257 with IMRT to a total dose of 79.2 Gy. For grade 2 and higher acute gastrointestinal/genitourinary (GI/GU) toxicity, both univariate and multivariate analyses showed a statistically significant decrease in grade 2 and higher acute collective GI/GU toxicity for IMRT. There was a trend for a clinically meaningful reduction in late grade 2 and higher GI toxicity with IMRT. Guckenberger et al. [6] analyzed 150 prostate cancer patients treated with dose-escalated, moderately hypofractionated cone-beam CT based IG-IMRT with a simultaneous integrated boost (SIB) technique. Acute genitourinary (GU) toxicity grade 1–2 was observed in 85 % of the patients. Gastrointestinal (GI) toxicity was mild with more than 80 % of the patients free from any GI toxicity during follow-up. Two patients suffered from late grade 3 GI toxicity. The rate of GU toxicity grade 2 or higher was less than 10 % at 6–12 months but increased continuously to 22.4 % at 60 months; grade 3 GU toxicity remained below 5 % during follow-up. Crehange et al. [3] evaluated the impact of PTV reduction when delivering IG-IMRT for patients with prostate cancer. The median dose prescribed to the prostate was 78 Gy (range 74–78 Gy). The incidence of grade 2 late genitourinary toxicity was 7.0 % for patients with a CTV–PTV margin of 5 mm (*n* = 87) and 6.6 % for patients with a CTV–PTV margin of 10 mm (*n* = 78; *p* = 1.00). The incidence of grade 2 late gastrointestinal toxicity was 1.2 and 2.6 % (*p* = 0.38), respectively.

The limitations of tracking the prostate position using implanted fiducial prostate markers should be mentioned. Changes of the prostate shape, rotational changes of the prostate position [5, 24] as well as intrafractional errors are not assessed with this method. Intrafractional prostate motion variability and CTV–PTV margin recommendations reported in various series have been nicely summarized by Skarsgard et al. [18]. A more sophisticated approach to minimize the PTV and to optimize the dose distribution would be “Adaptive Radiotherapy” where an adaption of the dose distribution can be achieved by daily CT-based image guidance [1, 8] in combination with gating or tracking of the target, thereby, considering inter- and intrafractional changes [2].

## Conclusion

A high dose with surprisingly low acute toxicity can be applied with daily IG-IMRT using implanted fiducial prostate markers for the definitive external beam radiotherapy of localized prostate cancer. Daily image guidance is clearly superior compared to image guidance every other fraction concerning adequate target coverage with minimal margins.
